# Development and validation of a reliable LC–MS/MS method for quantitative analysis of usnic acid in *Cladonia uncialis*

**DOI:** 10.1186/s13104-019-4580-x

**Published:** 2019-08-30

**Authors:** Natalia Sveshnikova, Tao Yuan, Jamie M. Warren, Michele D. Piercey-Normore

**Affiliations:** 1 School of Science and the Environment, Grenfell Campus, Memorial University of NL, 20 University Drive, Corner Brook, NL A2H 5G4 USA; 2 School of Science and the Environment, Boreal Ecosystem Research Facility, Grenfell Campus, Memorial University, 20 University Dr, Corner Brook, NL A2H 5G4 USA

**Keywords:** Liquid chromatography tandem mass-spectrometry, LC–MS/MS, Secondary metabolites, Lichen, Usnic acid, *Cladonia uncialis*

## Abstract

**Objective:**

The purpose of this study was to develop and validate a specific and sensitive liquid chromatography tandem mass-spectrometry method for quantification of usnic acid concentration in the lichen, *Cladonia uncialis*, suitable for detection of relatively small fluctuations of usnic acid concentration in response to environmental changes.

**Results:**

The resulting method was fully validated according to international guidelines and demonstrated good selectivity and sensitivity with minor levels of a matrix effect and high accuracy.

## Introduction

Usnic acid (UA) is one of the most common and most studied lichen-specific secondary metabolites and is taxonomically widely distributed in species of *Cladonia*, *Usnea*, *Lecanora*, *Ramalina*, *Evernia*, members of the Parmeliaceae, and other lichen genera [1]. Isolated in 1844 [2] and studied ever since, it is still of interest in industry for its antibacterial, antiviral, antioxidant, anti-inflammatory, analgesic and even anticancer properties [1, 3–5]. The biological role of UA in lichens is considered to be species-specific [6], and may include UV protection for the photobiont [7], from herbivores [8, 9], and from fungal and bacterial pathogens [10, 11].

Under natural conditions, the accumulation of UA in lichens is thought to depend on environmental factors [7, 12–14] with seasonal variation [15, 16]. Methods of extraction and quantification of UA were reported depending on available equipment, research goals and required accuracy of the method, making it difficult to compare across studies. One of the most reliable modern analytical methods is Liquid Chromatography Tandem-Mass Spectrometry (LC–MS/MS), having advantages of both accurate identification and quantification of the substance in question. The use of LC-UV with MS in recent publications [17, 18] was not sensitive enough for detection of subtle variation in UA, was not validated, and were conducted on species with matrix interferences. The development of a standard method to detect UA may help to elucidate its ecological role. The goal of this study was to establish a method for reliable monitoring of subtle changes in concentration of UA, specifically for *Cladonia uncialis* (L.) F.H. Wigg. [19–21], from the natural lichen thallus and validation according to Bioanalytical Method Validation (US and EU [22–25]).

## Main text

### Materials and methods

#### Chemicals

All reagents used were of analytical or higher grade and purchased from Sigma Aldrich (Merck KGaA) unless otherwise stated. Usnic acid (UA) standard stock solution was prepared by solubilising 1 mg of UA in 1 mL of 100% acetonitrile.

#### Sample preparation

50 mg of the top 10 mm of dry *Cladonia uncialis* thallus was crushed with mortar and pestle, soaked in 10 mL of 100% acetonitrile, vortexed for 30 s and agitated at 150 rpm on the shaker (LSE Orbital Shaker, Corning™ LSE™) for 20 min at room temperature. This extraction was repeated four times with the same material to obtain residual UA. All extracts were pooled into one glass tube, and the combined volume was adjusted to 50 mL with 100% acetonitrile.

For the control, an extract from *Cladonia ochrochlora* (a non-UA-producing species) was prepared in the same manner to emulate the matrix effect.

#### LC–MS/MS procedure

LTQ XL™ Linear Ion Trap Mass Spectrometer (Thermo Scientific™) was used to conduct mass spectroscopy. Filtered (Whatman^®^ Mini-UniPrep^®^ G2, PTFE membrane, pore size 0.2 μm) samples were injected in a volume of 10 μL into a C8 LC column (Phenomenex, Luna^®^ 3 µm C8(2) 100 Å, LC Column 100 × 2 mm) and separated by UltiMate™ 3000 RSLCnano System. Chromatographic separation was performed at a flow rate of 0.2 mL/min using a gradient elution program, starting from 80% of eluent A (water with 0.1% formic acid, v/v) and gradually changing to 5% A and back over 40 min. Exact gradient parameters: 80% of eluent A/20% B (100% acetonitrile acidified with 0.1% formic acid (v/v)) for 5 min, gradual changing to 5% A/95% B over 23 min, 5% A/95% B for 5 min, return to 80% of eluent A/20% B over 7 min.

Mass spectrometry measurements were performed on an LTQ OrbiTrap XL MS (Thermo Fisher). Samples were introduced to MS via electrospray ionisation using the following conditions: sheath gas flow rate, 30 (arbitrary units); auxiliary gas, 5 (arbitrary units); ESI voltage, 4.0 (kV); capillary voltage, − 35 (V); capillary temperature, 275 (°C); and tube lens voltage, − 110 (V). The collected spectra were scanned over the mass/charge number (m/z) range of 155–2000 atomic mass units (Xcalibur version 4.0). MS spectra were generated by collision-induced dissociation of the metabolite ions at normalized collision energy of 35%.

#### Method validation

The LC–MS/MS method was validated with respect to the specificity, linearity and sensitivity, precision and accuracy, matrix effects and recovery.

#### Specificity

The Specificity test was conducted by comparing chromatograms of 6 matrix blanks (*C. ochrochlora* extracts without UA) with a blank spike (UA in 100% acetonitrile) and a matrix spike (*C. ochrochlora* extracts spiked with UA).

#### Linearity and sensitivity

Two types of calibration standards were used for assessment of linearity and sensitivity of the method: different concentrations of UA in a solvent (acetonitrile) only as blank standards, and the same concentrations of UA in a matrix solution (*C. ochrochlora* extracts) as matrix standards. The final calibration curves included three replicates per calibration point, and linearity was assessed by linear regression.

The calibration range was narrowed down from a broader initial diapason (chosen based on existing literature) by visual observations of 10 analytical runs. The Limit of Detection (LOD) and Limit of Quantification (LOQ) were calculated using the formulas recommended by the guidelines mentioned above:

$$ {\text{LOD}} = 3.3\;*\;{\text{SD}} $$

$$ {\text{LOQ}} = 10\;*\;{\text{SD}} $$

where SD is the standard deviation of the signal at the lowest point of the calibration curve.

#### Accuracy and precision

The intra- and inter-day accuracy and precision measurements were conducted using measurements of three concentrations of UA (within the calibration range) dissolved in a matrix (extract) on a single assay, repeated (with triplicates) three times within 1 week.

#### Matrix effect

The Matrix effect was determined by comparison of the retention time (Rt) and the level of MS signal of the representative blank matrix spiked with a predetermined amount of UA with those obtained for the corresponding amount of UA in the solvent (100% acetonitrile).

To assure the matrix match between *C. uncialis* and *C. ochrochlora*, a similar comparison was made using both matrix samples spiked with the same amount of UA. Samples used for measurements contained 20 µL of final extract per mL of acetonitrile.

#### Recovery

The recovery was determined by comparing MS response level of spiked samples pre- and post-extraction, according to SANCO guide, using the average result of 4 replicates. The recovery percentage was calculated by dividing the value for the MS response of spiked pre-extracted sample by that of post extracted sample.

### Results and discussion

**Fig. 1:**
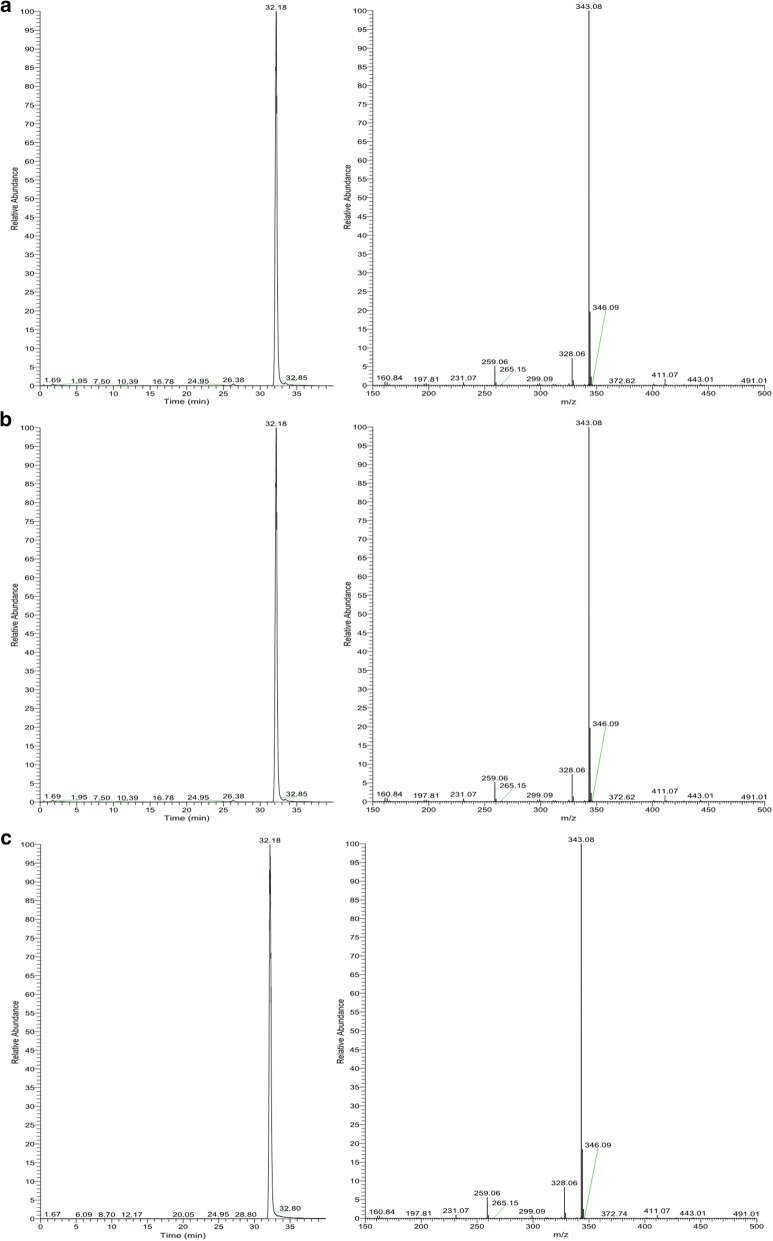
Mass spectra fragmentation pattern of UA mixed in or present in **a** acetonitrile, **b** *C. ochrochlora* matrix, **c** *C. uncialis* samples

Negative electrospray ionization mass spectrometry has been used for analysis of UA for some time [26, 27], resulting in an established fragmentation pattern with major ion m/z 343.08 and minor daughter ions at m/z 328.06 and m/z 259.08 (Spectrum BML00262 in MassBank of North America, SPLASH: splash10-0006-0009000000-eba42546783e6d051377). The same pattern was observed in our experiments (Fig. 1).

Optimisation of the LC–MS/MS protocol was directed toward obtaining distinctive symmetrical peaks combined with good resolution. The C8(2) LC column with 3 µm particle size run at a speed of no more than 0.25 mL/min with acetonitrile buffered with 0.1% of formic acid as a mobile phase demonstrated the best results for LC separation and provided the most favorable conditions for MS/MS measurements.

#### Matrix effect

Since some degree of matrix-induced ionisation suppression was previously reported [17], the modified matrix-matched calibration approach, proposed by the SANCO guide, was chosen as the most reliable for quantification analysis.

**Fig. 2:**
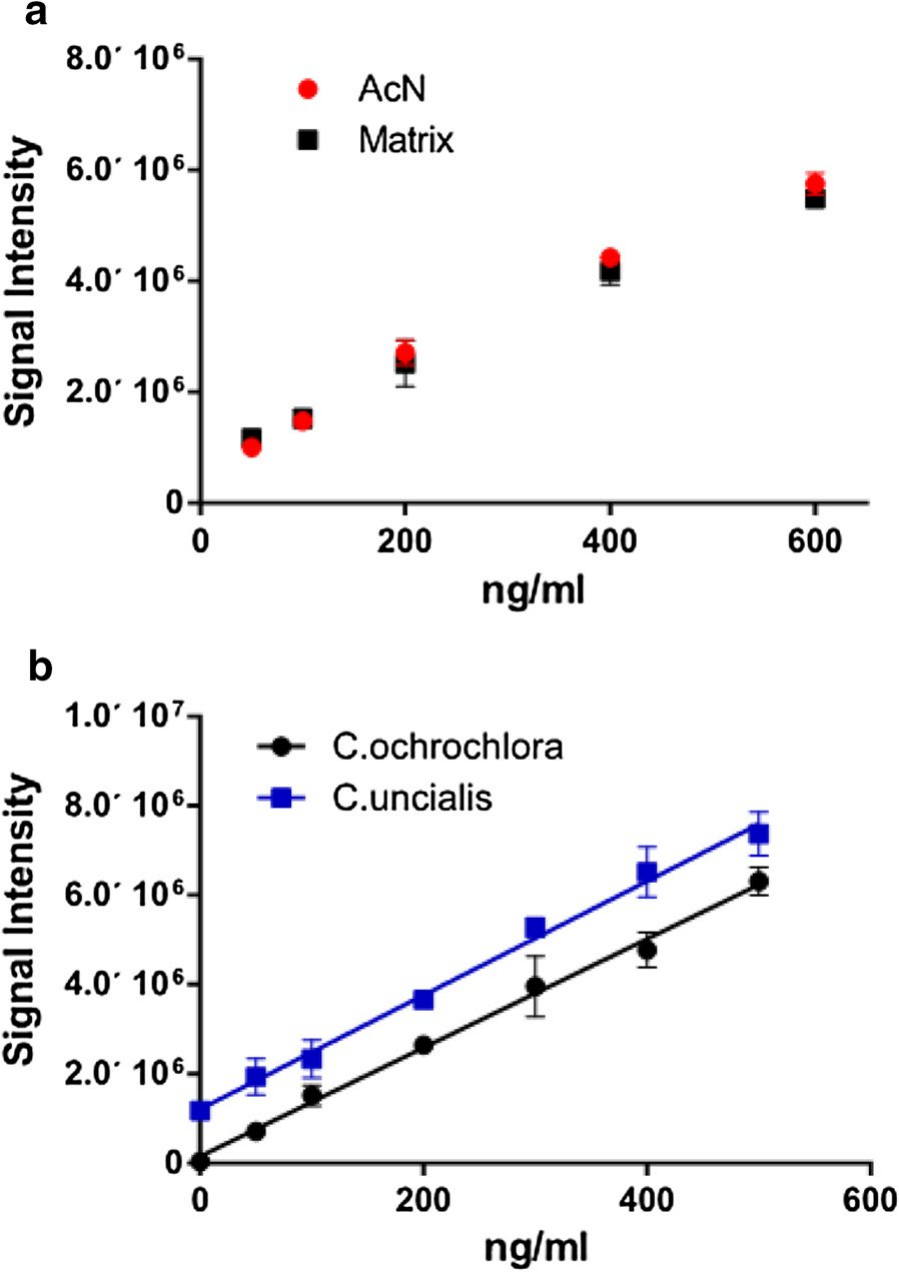
**a** Standard additions data recorded for acetonitrile (AcN) or extract of *C. ochrochlora* (Matrix). MS/MS response at m/z 343.08 was tested in three-five replicates at every indicated concentration (error bars indicate SD for every measuring point). **b** Calibration curves made for extracts of *C. ochlochlora* (Y = 12,164 * X + 1.54e + 005 (with R2 = 0.9428)) and *C. uncialis* (Y = 12,761 * X + 1.21e + 006 (with R2 = 0.9197)) spiked with UA standards. Error bars indicate SD, calculated for every measuring point

Since a non-UA-containing extract from *C. uncialis* cannot be obtained, another species was chosen as a matching matrix source—*Cladonia ochrochlora*, a lichen of the same genus and habitat, and known to contain no UA [28, 29]. (No difference was observed in peak quality or size between blanks) (UA standard added to pure acetonitrile) and matrix (UA standard in acetonitrile with addition of the extract of *C. ochrochlora*) (Figs. 1 and 2a). The un-spiked extract of *C. uncialis* did not demonstrate any deviations in peak shape from that of the standard. To test if signal strength was affected in samples of *C. uncialis*, both matrices (extracts of *C. ochrochlora* and *C. uncialis*) were compared using standard addition calibration curves created by spiking those extracts with known concentration of UA (Fig. 2b), and using four replicas for each concentration value.

Although not identical due to presence of UA in the *C. uncialis* samples (translated into Y-intercept shift), the calibration curves demonstrated no significant difference in slopes.

This supported the use of *C. ochrochlora* extract as a matching matrix for future experiments. The insignificance of the matrix effect on quality of the signal in our experiments in contrast to the matrix effect observed in other laboratories [17] could be explained by differences in either extract composition of analysed species, methods specifications, equipment used in experiments, sample preparation, or a combination of factors.

#### Specificity, linearity and sensitivity

A comparison of blanks (acetonitrile, matrix *C. ochrochlora* extracts without UA) with samples and spiked blanks (UA added to acetonitrile or matrix solution) demonstrated a high specificity of the method. Neither pure acetonitrile, nor pure matrix chromatograms showed any peaks with the UA fragmentation pattern, while peaks with the UA specific MS fragmentation were observed in samples and spiked blanks at very low (< 2 ng/mL) concentrations.

While UA indeed could be detected at very low concentrations (< 2 ng/mL), the linearity range was more restrictive: after a series of adjustments the reliable measuring interval fell between 50 and 500 ng/mL. The Limit of Detection (LoD) and Limit of Quantification (LoQ) were calculated using the calibration curve obtained for *C. ochrochlora* extract and was 2.2 ng/mL (LoD) and 32.3 ng/mL (LoQ).

The concentration of the UA sample extracted, using the described procedure with 50 mL final volume of the extract, is expected to be recovered within the quantitative linear range since concentrations of UA in *Cladonia* species under natural conditions vary between 0.4 and 3.8 in dry weight percentages, corresponding to 80–760 ng/mL in our experimental conditions [17, 30–32]. In the case of the UA concentration exceeding the suggested limits, an appropriate dilution was used.

#### Recovery

Recovery, a characteristic of extraction efficiency, was measured by comparison of UA-spiked samples where the known concentration of UA (2.5 µg to the final 50 mL volume) was added after or before the extraction and was 94%, consistent with that of Roach et al. [17]. Since three independent measurements produced similar results, and considering previous reports about a small fraction of UA ineradicable from the cell wall matrix [7, 33], the extraction efficiency was stable and high enough (about 94%) for further experiments.

#### Accuracy and precision

Precision was expressed as Coefficient of Variation (CV) and accuracy was expressed as Relative Error (RE), and were evaluated for three concentrations of UA within the linearity range: 50 ng/mL, 200 ng/mL and 500 ng/mL. The resulting fluctuations did not exceed 7% for Intra-assays and 11% for Inter-assays for CV, and 7% for RE calculations in both type of measurements (Table 1).

**Table 1:** Intra- and inter-assays accuracy and precision

Assay type	UA, ng/mL	CV, %	RE, %
Intra-assay	50	6.00	5.76
	200	3.88	1.43
	500	6.38	6.63
Inter-assay	50	4.48	6.48
	200	10.65	2.24
	500	9.98	1.26

Although deviations for accuracy and precision were within limits recommended by most of the guidelines (IUPAC, FDA and SANCO, where 15–20% is given as an acceptable level of variation), it is recommended that the calibration samples be included in every sequence in future experiments with re-evaluation of calibration graphs to negate the natural instability of signal in MS.

#### Stability

The matrix solution (extract of *C. ochrochlora*) spiked with UA at the concentration of 400 ng/mL, was kept at room temperature in darkness for 1 month and assessed five times during that period. Inter-assay RE was 2.31%, which is an acceptable level of deviation, demonstrating stability of UA under experimental conditions in this study.

### Conclusion

The method suggested in the present article proves to be suitable for accurate measurements of UA concentration in dried field samples of *Cladonia uncialis* under the conditions in this study: sample preparation and storage, maintaining concentration of UA in the extracts between 50 and 500 ng/mL, and regular adjustments of calibration.

## Limitations

The method presented in this paper was performed in a single laboratory and the validation was performed on a single species.

## Data Availability

All data generated or analysed during this study are included in this published article or can be obtained upon request to NS.
